# Serum concentration of extracellular cold-inducible RNA-binding protein is associated with respiratory failure in COVID-19

**DOI:** 10.3389/fimmu.2022.945603

**Published:** 2022-07-29

**Authors:** Felix Schagatay, Klara Diamant, Mats Lidén, Alicia Edin, Simon Athlin, Olof Hultgren, Clas Ahlm, Mattias N. E. Forsell, Johanna Savilampi, Johan Normark, Anna Lange, Sara Cajander

**Affiliations:** ^1^ Department of Infectious Diseases, CKF Region Västmanland, Västerås Hospital, Västerås, Sweden; ^2^ School of Medical Sciences, Örebro University, Örebro, Sweden; ^3^ Department of Radiology, Faculty of Medicine and Health, Örebro University, Örebro, Sweden; ^4^ Department of Surgical and Perioperative Sciences, Umeå University, Umeå, Sweden; ^5^ Department of Laboratory medicine, Faculty of Medicine and Health, Örebro University, Örebro, Sweden; ^6^ Department of Clinical Microbiology, Umeå University, Umeå, Sweden; ^7^ Department of Anaesthesiology and Intensive Care, Örebro University, Örebro, Sweden; ^8^ Department of Infectious Diseases, Faculty of Medicine and Health, Örebro University, Örebro, Sweden

**Keywords:** COVID-19, DAMPs, CIRP, eCIRP, inflammation, severity

## Abstract

Uncontrolled release of damage-associated molecular patterns (DAMPs) is suggested to be a major trigger for the dysregulated host immune response that leads to severe COVID-19. Cold-inducible RNA-binding protein (CIRP), is a newly identified DAMP that aggravates inflammation and tissue injury, and induces respiratory failure in sepsis. Whether CIRP contributes to the pathogenesis of respiratory failure in COVID-19 has not yet been explored.

**Aim:**

To investigate if the concentration of extracellular CIRP (eCIRP) in serum associates with respiratory failure and lung involvement by chest computed tomography (CT) in COVID-19.

**Methods:**

Herein we report a prospective observational study of patients with COVID-19 included at two University Hospitals in Sweden between April 2020 and May 2021. Serum from hospitalized patients in Örebro (N=97) were used to assess the association between eCIRP and the level of respiratory support and its correlation with pulmonary involvement on chest CT and inflammatory biomarkers. A cohort of hospitalized and non-hospitalized patients from Umeå (N=78) was used as an external validation cohort. The severity of disease was defined according to the highest degree of respiratory support; mild disease (no oxygen), non-severe hypoxemia (conventional oxygen or high-flow nasal oxygen, HFNO <50% FiO2), and severe hypoxemia (HFNO ≥50% FiO2, mechanical ventilation). Unadjusted and adjusted linear regression was used to evaluate peak eCIRP day 0-4 in respect to severity, age, sex, Charlson comorbidity score, symptom duration, and BMI.

**Results:**

Peak eCIRP concentrations were higher in patients with severe hypoxemia and were independently associated with the degree of respiratory support in both cohorts (Örebro; *p*=0.01, Umeå; *p*<0.01). The degree of pulmonary involvement measured by CT correlated with eCIRP, r_s_=0.30, *p*<0.01 (n=97).

**Conclusion:**

High serum levels of eCIRP are associated with acute respiratory failure in COVID-19. Experimental studies are needed to determine if treatments targeting eCIRP reduces the risk of acute respiratory failure in COVID-19.

## 1 Introduction

The disease COVID-19 encompasses a wide clinical spectrum, ranging from asymptomatic disease, or mild upper airway respiratory symptoms, to severe pneumonitis, respiratory failure and death (1). Before the introduction of vaccines against COVID-19, approximately 15% of symptomatic patients progressed to severe disease, which required respiratory support, and 5% of patients subsequently developed critical disease including acute respiratory distress syndrome (ARDS), septic shock or multiorgan failure (2). Advanced age, lack of SARS-CoV-2 antibody immunity, obesity, male sex, and the number of chronic comorbidities are today identified as the most important risk factors for patients to develop severe respiratory failure and death (3–6). Although a dysregulated immune response with delayed viral control and subsequent deleterious hyperinflammation is suggested to be a main feature of the pathogenesis in COVID-19, the mediators and culprit mechanisms of the hyper-inflammation are still not elucidated (7).

Previous studies and reviews of COVID-19 have suggested that damage-associated molecular patterns (DAMPs), a heterogenous group of proteins or nucleic acid molecules released from damaged cells that trigger the innate immune response, may play a key role in the development of severe COVID-19 (8, 9).

In this study we hypothesized that the extracellular form of cold-inducible RNA-binding protein (CIRP), i.e. eCIRP, a newly identified DAMP, may be a driver of COVID-19 associated respiratory failure as experimental studies have shown that it is an important mediator of sepsis induced acute lung injury (10) and bleomycin induced pulmonary fibrosis (11). Cellular stressors such as hypothermia and hypoxia are known to induce upregulation and release of eCIRP into the extracellular space and circulation (12–14). When recombinant CIRP is injected *in vivo*, it is shown to stimulate the release of tumor necrosis factor-α (TNF-α), interleukin 6 (IL-6), and high mobility group box 1 (HMGB1) from macrophages, and to cause tissue injury (12). eCIRP binds to the toll like receptor 4-myeloid differentiation factor 2 (TLR4-MD2) complex or directly to TLR4 on the surface on immune cells, epithelial and endothelial cells, and subsequently activates various downstream inflammatory pathways (15). Elevated serum and tissue levels of eCIRP have been reported in both systemic, organ-specific ischemic, and chronic inflammatory diseases (15). When first described serum levels of eCIRP were undetectable in healthy volunteers, while increased in patients with hemorrhagic shock admitted to the intensive care unit (ICU) (12). Later, serum eCIRP levels have been associated with severe disease and poor prognosis in patients with sepsis, community-acquired pneumonia, and acute pancreatitis (16–19). Extracellular cold-inducible RNA-binding protein knock out mice (eCIRP-/-) are protected against inflammatory disease (15, 20), and neutralizing antibodies against eCIRP are shown to attenuate inflammation and improve survival in animal models of hemorrhagic shock, sepsis, and hepatic ischemia (12, 21). Consequently, eCIRP has been proposed as a therapeutic target in several inflammatory diseases.

The role of eCIRP in the pathogenesis of COVID-19 have not yet been explored. In this study we aimed to evaluate serum concentrations of eCIRP in relation to the degree of respiratory support, the extent of lung involvement on chest computed tomography (CT), and inflammatory markers in patients with COVID-19.

## 2 Materials and methods

### 2.1 Study population and setting

Between April 2020 and May 2021, a prospective two-center observational study of patients with COVID-19 was conducted at the Departments of Infectious Diseases at Örebro University Hospital (study site 1) and Umeå University Hospital (study site 2). Hospitalized patients ≥ 18 years old (study site 1 and 2) and non-hospitalized patients ≥ 15 years old (study site 2), with PCR-verified (nasopharyngeal swab) SARS-CoV-2 infection, were eligible for study inclusion. Exclusion criteria were inability to adhere to study protocol, for example not being able to communicate in Swedish.

The patients were divided into three severity groups based on maximal respiratory support during hospital stay. Mild disease was defined as non-hospitalized patient, or hospitalized patient with no need of oxygen treatment. Non-severe hypoxemia was defined as patients treated with conventional oxygen, or high flow nasal oxygen (HFNO) treatment with fraction of inspired oxygen (FiO_2_) <50%. Severe hypoxemia was defined as treatment with HFNO with FiO_2_ ≥ 50%, or mechanical ventilation including extracorporeal membrane oxygenation (ECMO). In the present study eCIRP concentrations were primarily assessed in relation to the level of respiratory support, concentration of inflammatory markers and the maximal parenchymal infiltration on chest CT during hospitalization in samples from hospitalized patients enrolled in Örebro, study site 1. FiO_2_ ≥ 50% was chosen as a cut-off level to define patients with severe hypoxemia due to local guidelines recommending clinical assessment by intensive care specialists at this level of oxygen support. Patients enrolled at study site 2 (Umeå) were used as an external validation cohort to test/verify results from study site 1. Study site 2 cohort included both hospitalized and non-hospitalized patients.

### 2.2 Ethics

Ethical approval was achieved from the Swedish Ethical Review Authority, file number 2020-01557. A written informed consent was obtained from patients, or, if unable to consent, next of kin.

### 2.3 Study protocol

Hospitalized patients were sampled for blood every other day during the length of their hospital stay. These patients followed a study protocol with scheduled visits and tests 2 weeks, 1, 2, 3 months, 6 months and yearly for 5 years after hospital discharge. Non-hospitalized patients were sampled once on study inclusion and were thereafter followed according to the same blood sampling protocol as hospitalized patients.

At study site 1 (Örebro) structured chest CT assessment of radiological lung involvement was part of the regional clinical routine in the evaluation of hypoxemic COVID-19 patients on hospital admission and was often repeated if patients deteriorated (22). Serum samples from hospitalized patients were collected on days 0 (day of enrollment), 2, and 4, and from non-hospitalized patients on day 0, and analyzed for eCIRP concentrations. Biochemical tests, including C-reactive protein (CRP), IL-6, lymphocyte count and ferritin, were analyzed with routine laboratory methods at the different study sites.

Background data and clinical data were extracted from medical patient records.

### 2.4 Assessment of lung parenchymal involvement

The maximal extent of lung volume radiographic involvement during the hospital stay, was determined using the Örebro Covid-19 Scale (ÖCoS) (22). ÖCoS severity score ranges from 1-5, corresponding to the percentage of lung parenchymal involvement on chest CT, ÖCoS 1 < 10%, ÖCoS 2 = 10-25%, ÖCoS 3 = 25-50%, ÖCoS 4 = 50-75%, and ÖCoS 5 > 75%. In 14 patients the ÖCoS grading on chest CT was missing and was therefore graded retrospectively by a radiology specialist (M.L).

### 2.5 ELISA analyses

Cold-inducible RNA-binding protein concentrations in serum were analyzed in duplicate at 1:2 and 1:4 dilution with Human cold-inducible RNA-binding protein (CIRBP) ELISA kit, Cusabio, China, detection range 12.5-8000 pg/mL, according to the manufacturer’s instructions.

### 2.6 Statistical methods

Statistical analyses were performed using IBM SPSS for Windows, version 25 (IBM Corp., Armonk, NY, USA) and GraphPad PRISM, version 9.0.0 (GraphPad Software, San Diego, CA, USA). eCIRP concentrations showed non-normal distribution.

Baseline characteristics were compared between the severity groups using Chi Square, Fischer’s exact test and Kruskal Wallis test, as applicable.

The highest concentration of eCIRP (peak concentration) on day 0-4 after enrollment was determined for hospitalized patients. Unadjusted and adjusted linear regression with log10 eCIRP concentration (day 0 for non-hospitalized and peak value day 0-4 for hospitalized patients) as outcome variable was performed. Independent variables were maximal respiratory support (reference mild disease) and baseline factors age, sex, body max index (BMI), multiple comorbidities evaluated with Charlson comorbidity score (CCS), and symptom duration (days) on study enrollment (day 0). The results are presented as *B* and 95% confidence intervals (CI).

Spearman’s correlation was used to assess the association between peak eCIRP concentrations day 0-4 and ÖCoS-severity score, and the association between day 0 eCIRP and day 0 inflammatory markers (IL-6, ferritin, CRP, and lymphocyte count). The results are presented as Spearman’s rho (rs).

Statistical significance was set at *p*< 0.05.

## 3 Results

### 3.1 Characteristics of the study population

Baseline characteristics of the study cohorts are presented in **Table 1**. Ninety-seven (97) hospitalized patients were enrolled at site 1 (Örebro) of which 91 patients had at least one serum sample from day 0-4 available for eCIRP analysis. At site 2 (Umeå), 37 hospitalized and 41 non-hospitalized patients who had donated at least one serum sample from day 0-4 were included in the site 2 validation cohort.

**Table 1:** Demographics and clinical characteristics of study population.

	Örebro cohort			Umeå cohort		
	Mild disease^1^	Non-severe hypoxemia^2^	Severe hypoxemia^3^	Mild disease^1*^	Non-severe hypoxemia^2^	Severe hypoxemia^3^
	(*n*=12)	(*n=*48)	(*n=*31)	(*n=*49)	(*n=*19)	(*n=*8)
Age median (IQR)	57 (46-67)	53 (42-69)	59 (48-65)	52 (45-64)	52 (43-65)	60 (53-64)
Sex male	8 (67)	29 (60)	21 (68)	25 (51)	10 (53)	7 (88)
Hospital stay days median (IQR)	4 (2-5)	5 (4-7)	12 (8-19)	2 (2-5)#	5 (4-8)¤	16 (9-20)¤
Days since symptom onset median (IQR)^4^	9 (8-13)	11 (9-15)	11 (9-13)	7 (5-8)#	10 (7-12)	13 (8-17)
BMI median (IQR)	29.5 (28-31)	30 (27-34)	33 (28.5-37)	25 (23-28)	31 (28-37)	30 (27-32)
Active smoker	0	1 (2)¤	0¤	15 (30)	6 (32)	4 (50)
Charlson comorbidity score median (IQR)	0 (0-1)	0 (0-1)	0 (0-1)	0 (0-1)	0 (0-1)	0 (0-1)
Charlson comorbidity score ≥1	5 (42)	21 (44)	11 (36)	13 (27)	7 (37)	3 (38)
Hypertension	6 (50)	18 (38)	12 (39)	12 (25)	8 (42)	4 (8)
Heart disease	2 (17)	6 (13)	3 (10)	5 (10)	4 (21)	1 (13)
Chronic lung disease^a^	3 (25)	9 (19)	7 (23)	9 (18)	5 (26)	3 (38)
Diabetes	2 (18)	8 (17)	3 (10)	3 (6)	4 (21)	0
Immunosuppression	0	4 (8)	0	1 (2)	1 (5)	0
Chronic kidney disease	0	2 (4)	0	0	0	0

Data presented in table as n (%) if not further specified.

^1^Non-hospitalized patient, or hospitalized patient with no need of oxygen treatment.

^2^Conventional oxygen treatment, or high nasal flow oxygen (HFNO) treatment with FiO2 < 50%.

^3^HFNO with FiO2 ≥50%, mechanical ventilation, or extracorporeal membrane oxygenation (ECMO) treatment.

^4^at enrollment.

*Proportion of mild cases that was hospitalised 8/49 (16%).

a asthma, chronic obstructive pulmonary disease, pulmonal hypertension, lung fibrosis, or tuberculosis.

¤1 patient with missing data.

#2 patients with missing data.

At site 1 (Örebro), 12 patients were classified as having mild disease, 48 as having non-severe hypoxemia, and 31 as having severe hypoxemia. Three patients, one in the non-severe hypoxemia group, and two in the severe hypoxemia group died within 30 days. At site 2 (Umeå) 49 had mild disease, 19 had non-severe hypoxemia, and 8 had severe hypoxemia. Two patients who received HFNO treatment could not be classified with regards to respiratory disease severity due to a lack of information of the HFNO FiO2 settings. None of the patients at site 2 died within 30 days.

There was no significant difference between the severity groups regarding age, sex distribution, comorbidities (CCS), or BMI in any of the study cohorts. In the cohort at site 2 (Umeå), patients with severe hypoxemia and non-severe hypoxemia had significantly longer symptom duration before enrollment compared to patients with mild disease (both *p*<0.01), however in the cohort at site 1 (Örebro) no significant difference could be discerned. Patients with severe hypoxemia had significantly longer duration of hospital stay compared to patients with non-severe hypoxemia in both cohorts (both *p*<0.001), **Table 1**

### 3.2 Concentration of eCIRP is independently associated with disease severity

#### 3.2.1 Örebro cohort

**Figure 1:**
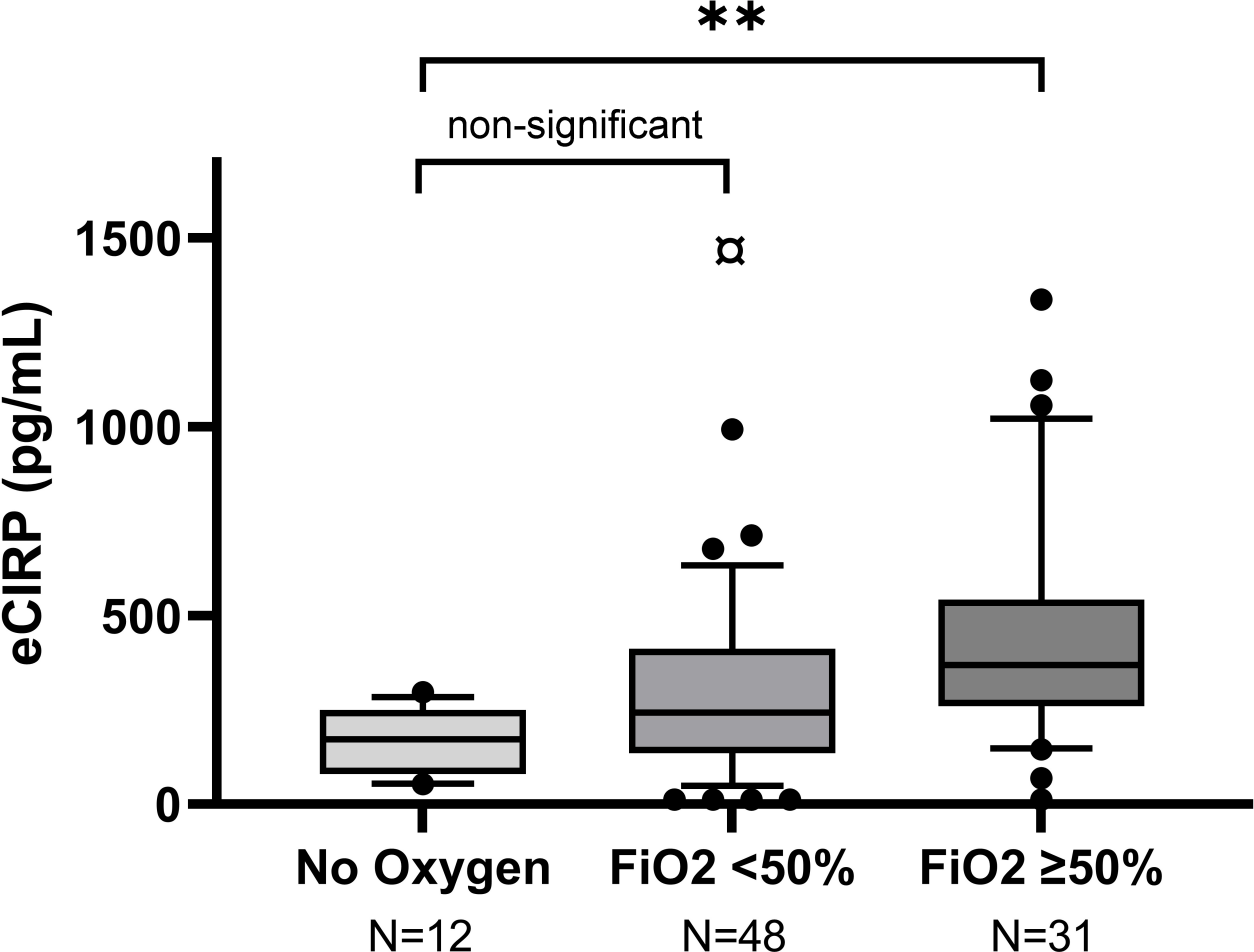
Peak concentrations of eCIRP in relation to COVID-19 severity in Örebro cohort. Box-plots representing the highest eCIRP concentrations on day 0-4 after enrollment, grouped by COVID-19 severity in Örebro cohort. Mild disease: No oxygen treatment. Non-severe hypoxemia: Conventional oxygen treatment or HFNC with FiO2 <50%. Severe hypoxemia: Treatment with HFNC with FiO2 ≥50% or mechanical ventilation. ¤ Outlier (2608 pg/mL), **p=0.01.

Severe hypoxemia was statistically significantly associated with higher mean peak eCIRP day 0-4 compared to mild disease, in the unadjusted and adjusted linear regression analyses (both *p*=0.01), **Figure 1**, **Table 2**. The association between severe hypoxemia and higher eCIRP remained when body mass index (BMI) was added to the adjusted model (*B* = 0.362, 95% CI 0.076-0.65). The association between non-severe hypoxemia and higher eCIRP was not statistically significant (unadjusted *p*=0.29, adjusted *p*=0.15), **Figure 1**.

**Table 2:** Linear regression with peak log_10_CIRP on day 0 to 4.

			Unadjusted		Adjusted		
	N (%)	B	95% CI	p-value	B	95% CI	p-value
**Örebro cohort**							
**Mild disease^1^**	12 (13)	ref	ref		ref	ref	ref
**Non-severe hypoxemia^2^**	48 (53)	0.15	-0.13 to 0.43	0.29	0.20	-0.07 to 0.46	0.15
**Severe hypoxemia^3^**	31 (34)	0.37	0.075-0.66	**0.01**	0.36	0.08-0.64	**0.01**
Age years*	56 (45-66)	0.008	0.001-0.014	**0.02**	0.009	0.003-0.15	**<0.01**
**Women**	33 (36)	ref	ref	ref	ref	ref	ref
**Men**	58 (64)	0.18	-0.14 to 0.37	0.07	0.21	0.003 to 0.02	**0.02**
**Charlson score^2^**	0 (0-1)*	0.038	-0.05 to 0.12	0.39	0.03	-0.06 to 0.11	0.55
**Symtom duration^3^**	11 (9-14)*	-0.018	-0.04 to 0.01	0.20	-0.02	-0.05 to 0.002	0.07
**Umeå cohort**							
**Mild disease**	49 (63)	ref	ref	ref	ref	ref	ref
**Non-severe hypoxemia**	19 (24.5)	0.30	0.08 to 0.52	**<0.01**	0.36	0.13 to 0.60	**<0.01**
**Severe hypoxemia**	8 (10.5)	0.74	0.43 to 1.05	**<0.01**	0.80	0.45 to 1.1	**<0.001**
Age years*	54 (46-64)	0.009	0.002 to 0.02	**0.02**	0.01	-0.001 to 0.01	0.07
**Women**	34 (44)	ref	ref	ref	ref	ref	ref
**Men**	44 (56)	0.12	-0.10 to 0.33	0.28	0.01	-0.17 to 0.22	0.77
**Charlson score^4^**	0 (0-1)*	0.052	-0.11 to 0.21	0.52	0.01	-0.14 to 0.15	0.94
**Symtom duration^5^**	7.5 (6-10)*	0.001	-0.02 to 0.02	0.94	-0.12	- 0.03 to 0.01	0.19

^1^Non-hospitalized patient, or hospitalized patient with no need of oxygen treatment.

^2^Conventional oxygen treatment, or high nasal flow oxygen (HFNO) treatment with FiO2 < 50%.

^3^HFNO with FiO2 ≥50%, mechanical ventilation, or extracorporeal membrane oxygenation (ECMO) treatment.

^4^per unit.

^5^days since debut of symptoms at enrolment.

*median (IQR).

Bold p-values are statistically significant.

#### 3.2.2 Umeå cohort

**Figure 2:**
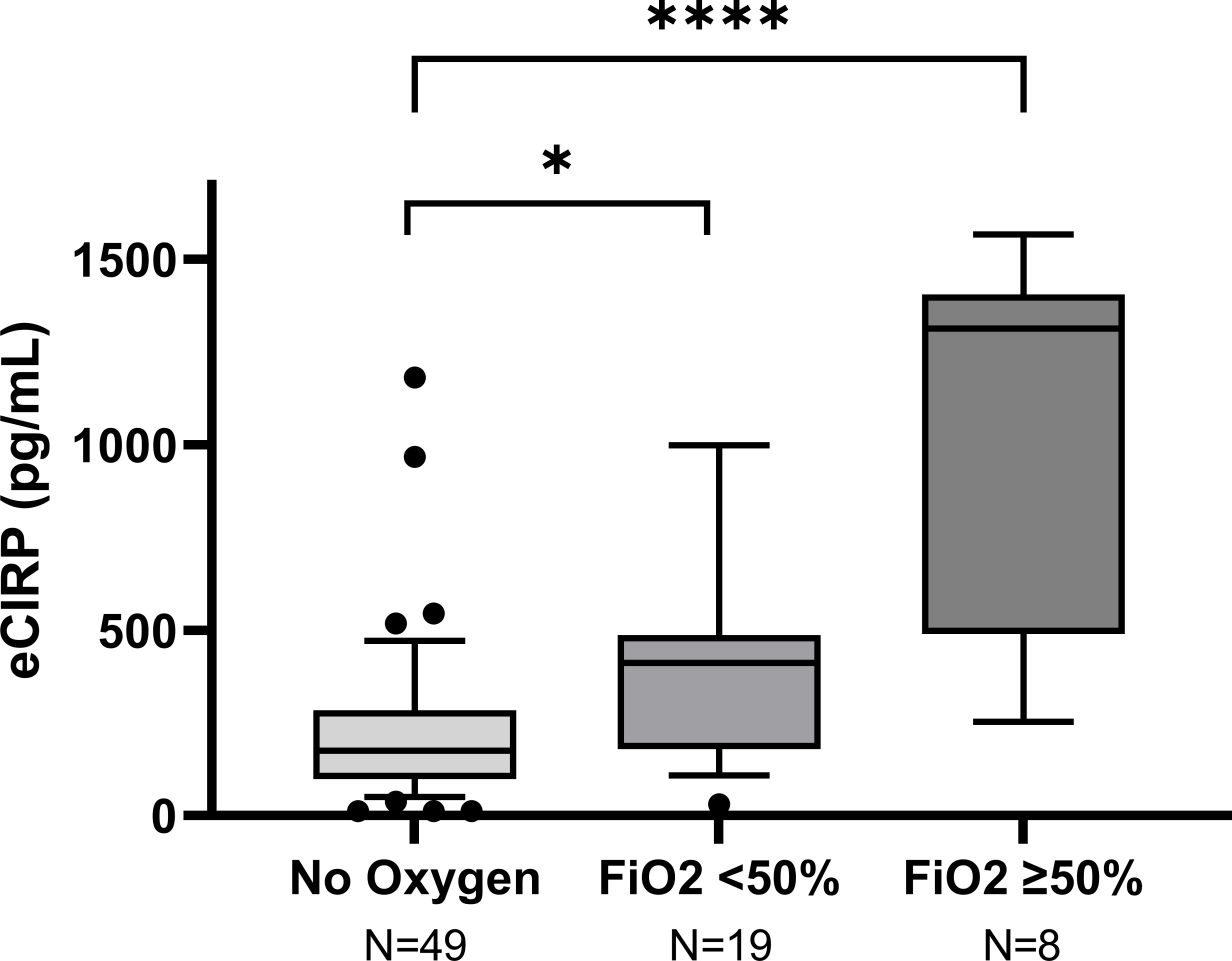
Peak concentration of eCIRP in relation to COVID-19 severity in the Umeå cohort. Box-plots representing the highest eCIRP concentrations on day 0-4 after enrollment, grouped by COVID-19 severity in Umeå cohort. Mild disease: No oxygen treatment. Non-severe hypoxemia: Conventional oxygen treatment or HFNC with FiO2 <50%. Severe hypoxemia: Treatment with HFNC with FiO2 ≥50% or mechanical ventilation. *p<0.001, ****p<0.001.

Both severe hypoxemia, and non-severe hypoxemia were statistically significantly associated with higher mean peak CIRP day 0-4 compared to mild disease in the unadjusted (both *p*<0.01) and adjusted (*p*<0.001 and <0.01 respectively) analyses, **Figure 2**; **Table 2**.

### 3.3 Peak eCIRP correlates with the extent of radiological lung infiltration

**Figure 3:**
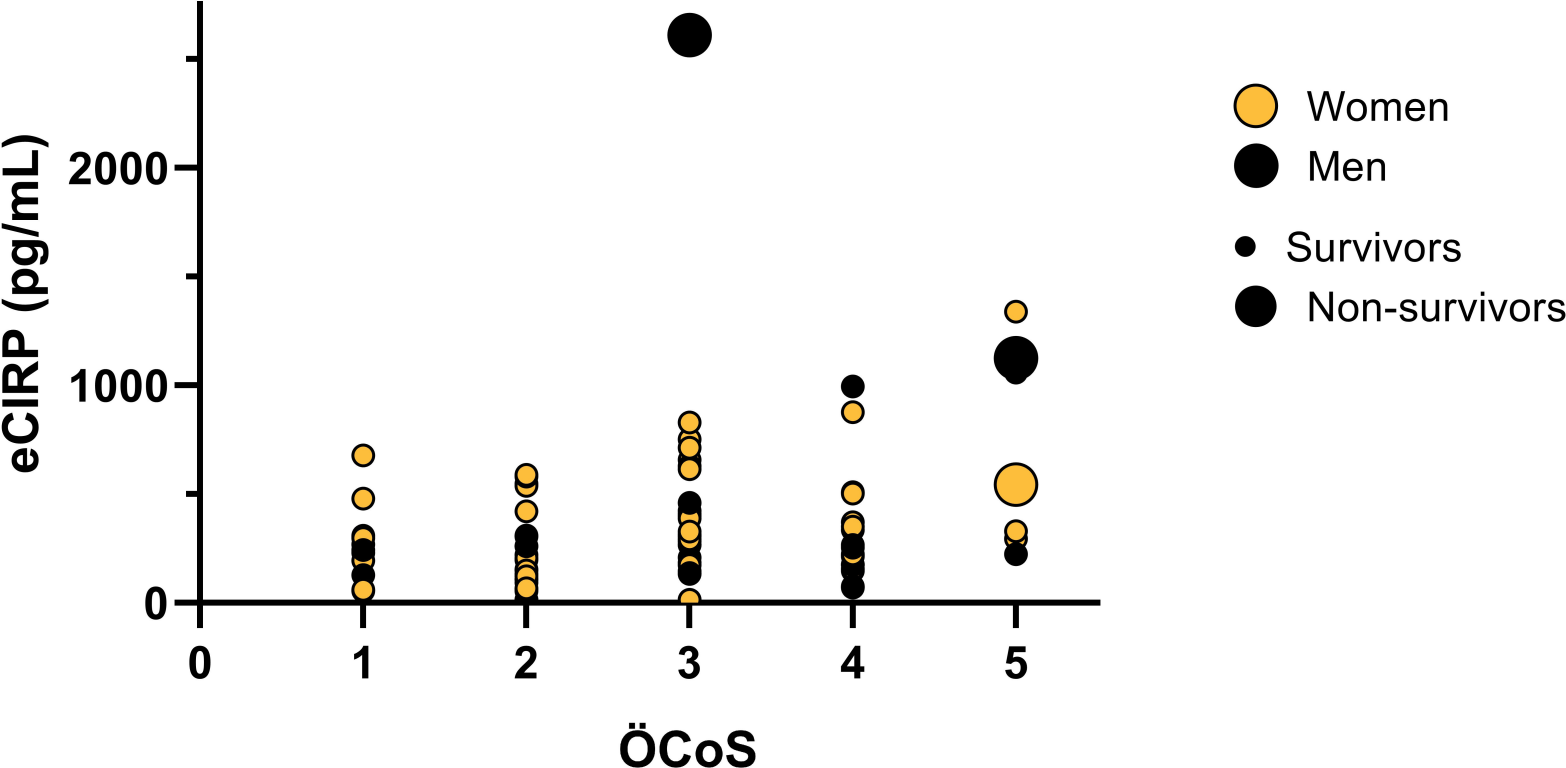
Peak eCIRP concentrations in relation to the extent of pulmonary infiltration on chest CT. Multivariate bubble-plot of eCIRP-values, Örebro Covid-19 Scale (ÖCoS), sex, and outcome. Peak concentrations of eCIRP measured 0-4 days after enrollment. Maximal parenchymal infiltration in percent visualized by chest CT during hospitalization, graded by ÖCoS. ÖCoS ranges from 1-5, corresponding to the percentage of lung parenchymal involvement, ÖCoS 1 < 10%, ÖCoS 2 = 10-25%, ÖCoS 3 = 25-50%, ÖCoS 4 = 50-75%, and ÖCoS 5 > 75%. Orange dots: Women. Black dots: Men. Large dots: Non-survivors (both men and women). Small dots: Survivors (both men and women).

Peak eCIRP concentrations day 0-4 were positively correlated to maximal extent of lung involvement assessed with ÖCoS-severity score (r_s_=0.30, *p*=0.005), **Figure 3**.

### 3.4 Concentration of eCIRP correlates to biomarkers of inflammation

Due to a high rate of missing biochemistry test data in the validation cohort (Umeå), we assessed eCIRP correlation to inflammatory markers exclusively in the cohort, site 1 (Örebro). Day 0 eCIRP concentrations were positively correlated to IL-6 (r_s_=0.308, *p*<0.01), ferritin (r_s_=0.306, *p*<0.01), CRP (r_s_=0.227, *p*=0.03), but there was no correlation to lymphocyte count (r_s_=-0.137, *p*=0.20), **Supplementary Figure 1**.

## 4 Discussion

Severe COVID-19 is characterized by a dysregulated, hyper-inflammatory host-response to infection. The proinflammatory mediator eCIRP shows promise as a treatment target in sepsis, but its role in COVID-19 has not yet been studied. In this study we show that serum concentration of eCIRP is associated with the severity of acute COVID-19. Patients who developed severe hypoxemia had higher eCIRP concentrations compared to patients with mild disease and the association remained after adjustment for age, sex, BMI, co-morbidities, and symptom duration. We confirmed the results in a validation cohort composed of a higher percentage of patients with mild disease, including non-hospitalized patients recruited at a separate study site. In the validation cohort, eCIRP concentrations also differed statistically between patients with mild disease and non-severe hypoxemia. Another important finding is that eCIRP correlates to parenchymal lung tissue involvement on chest CT, and to CRP, IL-6, and ferritin.

This is, to the best of our knowledge, the first study to report an association between eCIRP and respiratory failure in COVID-19. Only a small study of hospitalized patients in China (n=28), has previously studied eCIRP in COVID-19 (23). In contrast to the results of the present study, they found decreased plasma levels of eCIRP in patients with COVID-19 compared to controls (n=9), no association to severity, and a negative correlation to inflammatory cytokines (23). However, the limited sample size makes it difficult to draw firm conclusions from their study. Considering the evidence from studies on other hyper-inflammatory states and the known biology of eCIRP, it is plausible that serum concentrations would increase, not decrease, in COVID-19 patients with ongoing inflammation. Several previous preclinical and clinical studies have also demonstrated that hypoxia, both acute and chronic, induce upregulation of intracellular CIRP expression and that acute hypoxia result in release of eCIRP to the extracellular space and circulation (12, 13, 24). Also, in line with our results, pre-pandemic studies in sepsis and community acquired pneumonia, found that elevated eCIRP levels were prognostic for developing a severe disease or lethal outcome (16, 17).

In addition to an association to the level of hypoxemia, we found that eCIRP correlates to the visual extent of lung infiltration when measured by ÖCoS, a validated radiological chest CT scoring system (22). Bilateral ground-glass opacities, with or without signs of tissue consolidation, is the typical chest CT presentation of COVID-19. The extent of parenchymal infiltration, measured by chest CT on hospital admission is known to correlate to the duration of hospitalization, intensive care admission or death in COVID-19 (1, 22, 25). Patients with a high percentage of lung involvment (>75%) by the visal scoring scale by ÖCoS have a high likelihood of developing a critical disease due to respiratory failure (22). The correlation between eCIRP and ÖCoS therefore strengthens the hypothesis that eCIRP-driven inflammation contributes to development of respiratory failure of COVID-19.

Respiratory failure and severe hypoxemia in COVID-19 is known to be linked to a hyper-inflammatory, pro-coagulative state in the lungs, which causes a mismatch between ventilation and perfusion due to edema, fibrosis, and to activated lung endothelia with disseminated micro vasculopathy with thrombosis and bleeding. The injection of exogenous eCIRP into healthy mice has been shown to cause a similar picture with lung injury characterized by vascular leakage, edema, neutrophil infiltration, NLRP3 inflammasome activation, and local production of proinflammatory cytokines (TNF-α, IL-1β) (26). Moreover, eCIRP is shown to induce pulmonary fibrosis in mice, and to induce release of neutrophil extracellular traps (NETs) during sepsis, resulting in lung injury (27). In COVID-19, NET concentration has shown to be increased in both human serum and tissue, suggesting that it contributes to the development of severe disease. Although this study cannot address the mechanisms of eCIRP induced respiratory failure in COVID-19, the results from preclinical studies and pre-pandemic clinical studies demonstrate that CIRP itself can induce the same pathology that is characteristic for respiratory failure in COVID-19. Taken together, it is likely that eCIRP may aggravate inflammation and tissue injury leading to the development of severe respiratory failure in COVID-19. Future studies are required to define the pathways involved and if active or passive secretion is the leading mechanism of eCIRP secretion in COVID-19. In sepsis, active eCIRP secretion is shown to be linked to inflammasome activation, regulated by the gasdermin D (GSDMD) pathway (28). It is possible that the same pathway regulates eCIRP secretion in COVID-19 given that inflammasome activation with engagement of the GSDMD-pathway is shown to contribute to the pathology of COVID-19 (29). However, the lytical viral replication stage and necrosis may also lead to passive eCIRP release, further aggravating inflammation. Thus, it can be proposed that eCIRP is released by a combination of active and passive processes in COVID-19.

Extracellular CIRP was positively correlated to the pro-inflammatory biomarkers CRP, IL-6, and ferritin. Similar correlations between eCIRP and biomarkers for severity, i.e., CRP, IL-6, and procalcitonin (PCT) has been observed in sepsis, community acquired pneumonia, and acute pancreatitis (16–18). An association between eCIRP and non-COVID-19 induced inflammation has previously been established in a number of cells resident in pulmonary tissue, e.g., macrophages, alveolar epithelial cells, endothelial cells, and pulmonary fibroblasts (10, 12, 26, 30, 31).

This study has several limitations. Chest CT was not included in the prospective study design and therefore only possible to be assessed in hospitalized patients from site 1, where it was part of the clinical routine of hypoxemic patients (22). Moreover, inflammatory biomarkers sampled simultaneously as eCIRP were only possible to assess in patients from the cohort at site 1. The relatively small sample size of patients with mild disease at site 1 may therefore affect the generalizability of these correlations in a cohort with non-hypoxemic patients. Second, in contrast to previous studies on COVID-19, we had a greater proportion of older patients in the mild disease group, which may also affect the generalizability. However, age did not affect the concentrations of eCIRP in the present study. Third, we focused on samples with the highest concentrations on day 0-4, not from study inclusion only. This was possible due to the variation of symptom duration on study inclusion. To account for the effect of symptom duration on eCIRP expression, we include this as a covariate in the regression analysis.

The strength of the study is the prospective design with data on the levels of respiratory support and that the results are validated in a different cohort including a different case-mix, recruited at a separate study site.

## 5 Conclusion

In this two-center study, we conclude that high serum levels of eCIRP are associated with acute respiratory failure in COVID-19. Due to the observational design, we cannot draw firm conclusions about the role of eCIRP in the pathogenesis of COVID-19. Nevertheless, with the evidence from previous studies on sepsis, demonstrating that eCIRP is involved in the same inflammatory pathways that have been identified as cornerstones in the pathogenesis of COVID-19 induced acute respiratory failure, it is biologically plausible that it has a key function in the pathogenesis of severe COVID-19.

Future clinical, and experimental studies are required to define the mechanistic role of eCIRP in COVID-19. An important area of investigation would be to study how eCIRP affects NETosis and to determine if eCIRP antagonists protects from progression to severe respiratory failure in COVID-19.

## Data availability statement

The raw data supporting the conclusions of this article will be made available by the authors, without undue reservation.

## Ethics statement

The study involving human participants was reviewed and approved by the Swedish Ethical Review Authority, file number 2020-01557. Written informed consent to participate in this study was provided by the participants' legal guardian/next of kin.

## Author contributions

AL and SC were involved in overall planning and supervised the work. SC, CA, JN, and AE initiated and supported the prospective study and SA, SC, AL, JN, AE participated in patient enrollment. SC, AL, FS and KD processed the laboratory data, performed the statistical analysis, and designed the figures. ML performed ÖCoS grading of Chest CTs where the initial grading was missing. FS and KD drafted the manuscript. AE edited the figures. ML, JS, OH, SA and AE aided in interpreting the results. All authors critically revised the manuscript. All authors approved the submitted version of the manuscript.

## Funding

This work was supported by grants from Nyckelfonden Region Örebro County (DNR OLL-938628, OLL-961416) to OH and SciLifeLab National COVID-19 Research Program (VC-2020-0015), financed by the Knut and Alice Wallenberg Foundation, Umeå University & Region Västerbotten, Swedish Heart-Lung Foundation to CA and a consolidator grant from the Swedish Research Council (DNR 2020-06235) to MF and Centrum för klinisk forskning (CKF) Region Västmanland.

## Acknowledgments

We would like to thank patients and controls for their participation, and assistance from research and clinically working nurses, assistant nurses and other personnel in their help with the study during pressured times.

## Conflict of interest

The authors declare that the research was conducted in the absence of any commercial or financial relationships that could be construed as a potential conflict of interest.

## Publisher’s note

All claims expressed in this article are solely those of the authors and do not necessarily represent those of their affiliated organizations, or those of the publisher, the editors and the reviewers. Any product that may be evaluated in this article, or claim that may be made by its manufacturer, is not guaranteed or endorsed by the publisher.

## Supplementary material

Supplementary Figure 1 Heat map of correlations between inflammatory markers and eCIRP sampled on day 0. Color indicates correlation strength as demonstrated in the longitudinal bar.
