# Supplementary figures and images for: Serum concentration of extracellular cold-inducible RNA-binding protein is associated with respiratory failure in COVID-19
Source: Front Immunol. 2022 Jul 29;13:945603. doi: 10.3389/fimmu.2022.945603 (PMC9373926; doi:10.3389/fimmu.2022.945603)

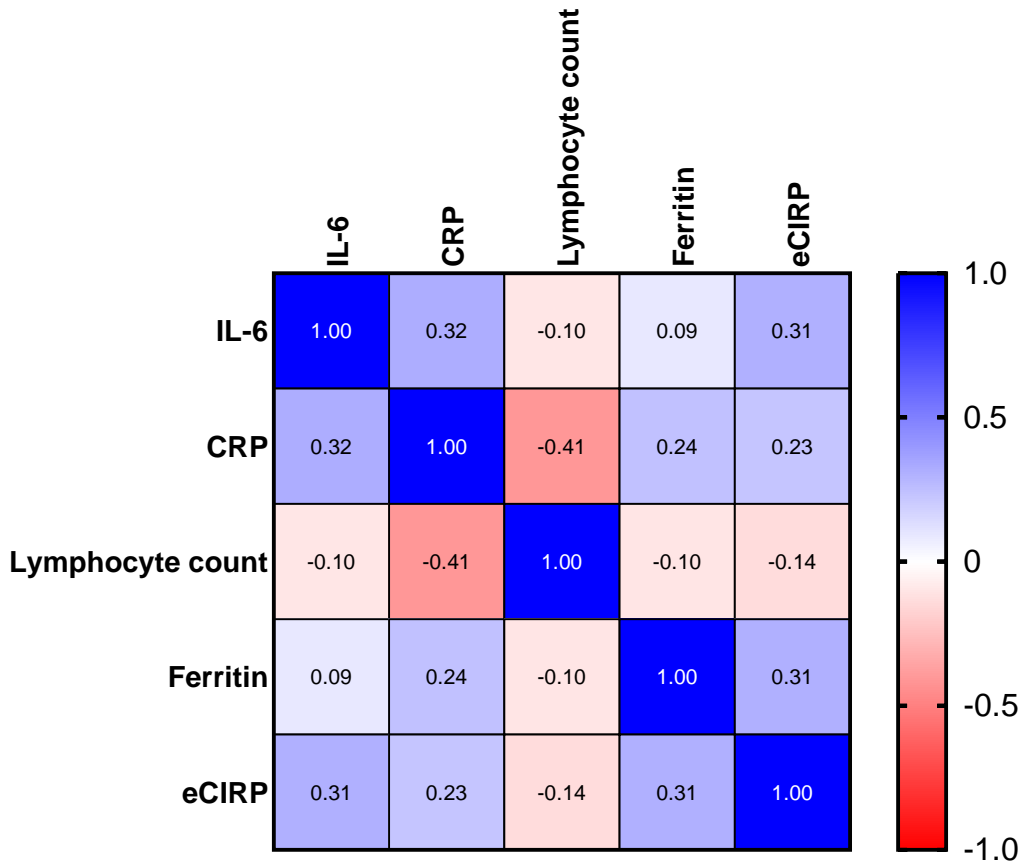

Supplement: Supplementary Figure 1 — Heat map of correlations between inflammatory markers and eCIRP sampled on day 0. Color indicates correlation strength as demonstrated in the longitudinal bar. [file Image_1.pdf]
